# *N*-(2-Ferrocenylethyl­idene)-4-(trifluoro­meth­yl)aniline

**DOI:** 10.1107/S1600536809011039

**Published:** 2009-03-28

**Authors:** Wolfgang Imhof

**Affiliations:** a Institute of Inorganic and Analytical Chemistry, Friedrich Schiller University, August-Bebel-Strasse 2, 07743 Jena, Germany

## Abstract

The title compound, [Fe(C_5_H_5_)(C_13_H_9_F_3_N)], was prepared by a condensation reaction from ferrocenylcarbaldehyde and 4-(trifluoro­meth­yl)aniline. The cyclo­penta­dienyl (Cp) rings are coplanar [dihedral angle = 1.4 (3)°] and the imine function is situated in the same plane. The aromatic substituent is bent out of the plane of the Cp ring to which the imine group is attached by 44.5 (4)°. The F atoms of the trifluoro­methyl substituent are disordered [occupancies 0.52 (2)/0.48 (2)].

## Related literature

For the structures of ferrocenylpropenal and imines derived from it, see: Imhof (1997 ▶, 1998 ▶, 2004 ▶, 2005 ▶).
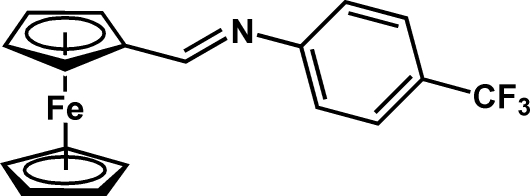


## Experimental

### 

#### Crystal data

[Fe(C_5_H_5_)(C_13_H_9_F_3_N)]
*M* *_r_* = 357.15
Triclinic, 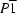

*a* = 5.8446 (7) Å
*b* = 10.383 (1) Å
*c* = 12.972 (2) Å
α = 100.467 (8)°
β = 91.670 (5)°
γ = 99.152 (8)°
*V* = 762.9 (2) Å^3^
*Z* = 2
Mo *K*α radiation
μ = 1.02 mm^−1^
*T* = 183 K
0.12 × 0.08 × 0.02 mm

#### Data collection

Nonius KappaCCD diffractometer
Absorption correction: none
2064 measured reflections
2064 independent reflections
1937 reflections with *I* > 2σ(*I*)
θ_max_ = 23.3°

#### Refinement

*R*[*F* ^2^ > 2σ(*F* ^2^)] = 0.035
*wR*(*F* ^2^) = 0.099
*S* = 0.79
2064 reflections
236 parameters
H-atom parameters constrained
Δρ_max_ = 0.25 e Å^−3^
Δρ_min_ = −0.41 e Å^−3^

### 

Data collection: *COLLECT* (Nonius, 1998 ▶); cell refinement: *DENZO* (Otwinowski & Minor, 1997 ▶); data reduction: *DENZO*; program(s) used to solve structure: *SHELXS97* (Sheldrick, 2008 ▶); program(s) used to refine structure: *SHELXL97* (Sheldrick, 2008 ▶); molecular graphics: *XP* (Siemens, 1990 ▶); software used to prepare material for publication: *XP*.

## Supplementary Material

Crystal structure: contains datablocks I, global. DOI: 10.1107/S1600536809011039/hg2490sup1.cif

Structure factors: contains datablocks I. DOI: 10.1107/S1600536809011039/hg2490Isup2.hkl

Additional supplementary materials:  crystallographic information; 3D view; checkCIF report

## Figures and Tables

**Fig. 1.**
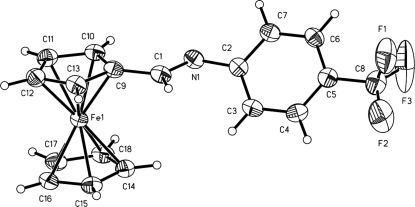
Molecular structure of the title compound with displacement ellipsoids at the 40% probability level.

## supplementary crystallographic information

### Comment

In the course of a study on imines derived from aldehydes and amines exhibiting ferrocenyl substituents we recognized that ferrocenyl-prop-2-enal as well as some of the corresponding imines crystallize in non-centrosymmetric space groups (Imhof, 1997, 1998, 2004, 2005). The title compound crystallizes in the centrosymmetric space group *P*1. All bond lengths and bond angles are of expected values. The Cp rings are coplanar (dihedral angle 1.4 (3)°) and the imine function is situated in the same plane. The aromatic substituent is bent out of the plane of the Cp the imine moiety is attached to by 44.5 (4)°. The shortest intermolecular distances are observed between fluorine atoms and hydrogen atoms of a Cp ring. Nevertheless, since the CF_3_ substituent is highly disordered these contacts should not be discussed as C–H···F hydrogen bonds.

### Experimental

500 mg ferrocenylcarbaldehyde (2.34 mmol) were dissolved in 20 ml of anhydrous ethanol together with an equimolar amount of 4-trifluoromethyl-aniline (376 mg) and 10 mg of *p*-toluenesulfonic acid. The solution was stirred at room temperature for 1 h. After evaporation of 15 ml of the solvent the remaining solution was put into the refrigerator at 277 K resulting in the precipitation of the title compound as crystalline material (yield: 600 mg, 72%). MS (EI) [m/*z*, %]: 357 (*M*^+^, 100), 338 (*M*^+^ - F, 11), 292 (*M*^+^ - Cp, 3), 236 (*M*^+^ - CpFe, 3), 216 (C_12_H_8_NF_2_^+^, 71), 186 (C_10_H_10_Fe^+^, 11), 167 (C_12_H_9_N^+^, 49, 121 (CpFe^+^, 19), 56 (Fe^+^, 13); ^1^H-NMR (CDCl_3_, 298 K) [p.p.m.]: 4.12 (s, 5H, Cp), 4.18–4.45 (m, 4H, Cp), 7.14–7.19 (m, 2H, CH_ar_), 7.58–7.61 (m, 2H, CH_ar_), 8.08 (m, 1H, CH=N).

### Refinement

Hydrogen atoms were positioned geometrically at distances of 0.95 Å for aromatic C—H functions and the imine C—H group and were refined riding on their parent atoms with isotropic thermal parameters of 1.2 times the corresponding values of their parent atoms.

### Crystal data

**Table:**

[Fe(C_5_H_5_)(C_13_H_9_F_3_N)]	*Z* = 2
*M**_r_* = 357.15	*F*(000) = 364
Triclinic, *P*1	*D*_x_ = 1.555 Mg m^−^^3^
Hall symbol: -P 1	Mo *K*α radiation, λ = 0.71073 Å
*a* = 5.8446 (7) Å	Cell parameters from 2064 reflections
*b* = 10.383 (1) Å	θ = 3.2–23.3°
*c* = 12.972 (2) Å	µ = 1.02 mm^−^^1^
α = 100.467 (8)°	*T* = 183 K
β = 91.670 (5)°	Plate, red
γ = 99.152 (8)°	0.12 × 0.08 × 0.02 mm
*V* = 762.9 (2) Å^3^	

### Data collection

**Table:**

Nonius KappaCCD diffractometer	1937 reflections with *I* > 2σ(*I*)
Radiation source: fine-focus sealed tube	θ_max_ = 23.3°, θ_min_ = 3.5°
graphite	*h* = 0→6
ω and φ scans	*k* = −11→11
2064 measured reflections	*l* = −14→14
2064 independent reflections	

### Refinement

**Table:**

Refinement on *F*^2^	Primary atom site location: structure-invariant direct methods
Least-squares matrix: full	Secondary atom site location: difference Fourier map
*R*[*F*^2^ > 2σ(*F*^2^)] = 0.035	Hydrogen site location: inferred from neighbouring sites
*wR*(*F*^2^) = 0.099	H-atom parameters constrained
*S* = 0.79	*w* = 1/[σ^2^(*F*_o_^2^) + (0.0772*P*)^2^ + 0.4666*P*] where *P* = (*F*_o_^2^ + 2*F*_c_^2^)/3
2064 reflections	(Δ/σ)_max_ = 0.003
236 parameters	Δρ_max_ = 0.25 e Å^−^^3^
0 restraints	Δρ_min_ = −0.41 e Å^−^^3^

### Special details

**Table:**

Geometry. All e.s.d.'s (except the e.s.d. in the dihedral angle between two l.s. planes) are estimated using the full covariance matrix. The cell e.s.d.'s are taken into account individually in the estimation of e.s.d.'s in distances, angles and torsion angles; correlations between e.s.d.'s in cell parameters are only used when they are defined by crystal symmetry. An approximate (isotropic) treatment of cell e.s.d.'s is used for estimating e.s.d.'s involving l.s. planes.
Refinement. Refinement of *F*^2^ against ALL reflections. The weighted *R*-factor *wR* and goodness of fit *S* are based on *F*^2^, conventional *R*-factors *R* are based on *F*, with *F* set to zero for negative *F*^2^. The threshold expression of *F*^2^ > σ(*F*^2^) is used only for calculating *R*-factors(gt) *etc*. and is not relevant to the choice of reflections for refinement. *R*-factors based on *F*^2^ are statistically about twice as large as those based on *F*, and *R*- factors based on ALL data will be even larger.

### Fractional atomic coordinates and isotropic or equivalent isotropic displacement parameters (Å^2^)

**Table:**

	*x*	*y*	*z*	*U*_iso_*/*U*_eq_	Occ. (<1)
Fe1	0.48698 (5)	0.24098 (3)	0.23674 (3)	0.03550 (19)	
C1	0.3215 (5)	0.3525 (3)	0.0515 (2)	0.0442 (6)	
H1	0.4421	0.3603	0.0043	0.053*	
N1	0.1114 (4)	0.3180 (2)	0.01264 (18)	0.0461 (6)	
C2	0.0736 (5)	0.2917 (3)	−0.0978 (2)	0.0420 (6)	
C3	0.2148 (5)	0.2243 (3)	−0.1663 (2)	0.0467 (7)	
H3	0.3436	0.1928	−0.1391	0.056*	
C4	0.1667 (5)	0.2035 (3)	−0.2739 (2)	0.0483 (7)	
H4	0.2636	0.1586	−0.3202	0.058*	
C5	−0.0220 (5)	0.2480 (3)	−0.3142 (2)	0.0463 (7)	
C6	−0.1655 (5)	0.3124 (3)	−0.2467 (2)	0.0467 (7)	
H6	−0.2961	0.3420	−0.2740	0.056*	
C7	−0.1173 (4)	0.3332 (3)	−0.1395 (2)	0.0463 (7)	
H7	−0.2167	0.3767	−0.0935	0.056*	
C8	−0.0774 (7)	0.2247 (4)	−0.4301 (3)	0.0617 (9)	
F1	−0.056 (3)	0.3334 (12)	−0.4661 (11)	0.101 (5)	0.52 (2)
F2	0.1063 (14)	0.1998 (13)	−0.4864 (8)	0.108 (4)	0.52 (2)
F3	−0.312 (3)	0.184 (3)	−0.4518 (9)	0.170 (8)	0.52 (2)
F1X	−0.140 (4)	0.3235 (14)	−0.4636 (12)	0.124 (6)	0.48 (2)
F2X	0.020 (5)	0.144 (2)	−0.4837 (9)	0.195 (9)	0.48 (2)
F3X	−0.225 (3)	0.1227 (9)	−0.4659 (7)	0.097 (4)	0.48 (2)
C9	0.3850 (4)	0.3802 (3)	0.1628 (2)	0.0417 (6)	
C10	0.2396 (5)	0.3601 (3)	0.2476 (2)	0.0440 (6)	
H10	0.0763	0.3315	0.2418	0.053*	
C11	0.3828 (5)	0.3903 (3)	0.3413 (2)	0.0492 (7)	
H11	0.3318	0.3855	0.4096	0.059*	
C12	0.6162 (5)	0.4291 (3)	0.3163 (2)	0.0494 (7)	
H12	0.7474	0.4546	0.3649	0.059*	
C13	0.6191 (5)	0.4234 (3)	0.2070 (2)	0.0463 (7)	
H13	0.7524	0.4443	0.1692	0.056*	
C14	0.4441 (5)	0.0678 (3)	0.1292 (2)	0.0520 (7)	
H14	0.3790	0.0548	0.0594	0.062*	
C15	0.6824 (5)	0.1108 (3)	0.1618 (2)	0.0477 (7)	
H15	0.8049	0.1312	0.1178	0.057*	
C16	0.7044 (5)	0.1177 (3)	0.2719 (2)	0.0455 (7)	
H16	0.8444	0.1435	0.3149	0.055*	
C17	0.4799 (5)	0.0790 (3)	0.3063 (2)	0.0498 (7)	
H17	0.4436	0.0748	0.3767	0.060*	
C18	0.3203 (5)	0.0479 (3)	0.2188 (3)	0.0503 (7)	
H18	0.1579	0.0187	0.2195	0.060*	

### Atomic displacement parameters (Å^2^)

**Table:**

	*U*^11^	*U*^22^	*U*^33^	*U*^12^	*U*^13^	*U*^23^
Fe1	0.0315 (3)	0.0357 (3)	0.0406 (3)	0.00535 (17)	0.00559 (16)	0.01043 (17)
C1	0.0399 (15)	0.0416 (15)	0.0553 (17)	0.0084 (12)	0.0093 (12)	0.0175 (12)
N1	0.0393 (13)	0.0502 (14)	0.0516 (14)	0.0081 (10)	0.0064 (10)	0.0159 (11)
C2	0.0398 (14)	0.0418 (16)	0.0456 (15)	0.0028 (12)	0.0052 (11)	0.0147 (12)
C3	0.0395 (14)	0.0425 (16)	0.0614 (18)	0.0096 (12)	0.0051 (13)	0.0160 (13)
C4	0.0481 (16)	0.0436 (16)	0.0538 (17)	0.0066 (13)	0.0155 (13)	0.0100 (13)
C5	0.0520 (16)	0.0393 (16)	0.0486 (16)	0.0025 (13)	0.0053 (12)	0.0147 (12)
C6	0.0414 (15)	0.0453 (16)	0.0550 (17)	0.0041 (12)	−0.0011 (12)	0.0174 (13)
C7	0.0376 (14)	0.0494 (17)	0.0545 (17)	0.0081 (12)	0.0080 (12)	0.0149 (13)
C8	0.079 (2)	0.053 (2)	0.0518 (18)	−0.002 (2)	0.0017 (18)	0.0162 (17)
F1	0.148 (8)	0.086 (7)	0.061 (4)	−0.032 (7)	−0.020 (4)	0.042 (4)
F2	0.082 (5)	0.202 (12)	0.053 (4)	0.038 (5)	0.032 (3)	0.040 (5)
F3	0.102 (7)	0.304 (19)	0.065 (5)	−0.074 (10)	−0.022 (4)	0.032 (9)
F1X	0.217 (15)	0.110 (11)	0.062 (5)	0.098 (10)	−0.029 (7)	0.008 (6)
F2X	0.39 (3)	0.175 (14)	0.056 (5)	0.198 (16)	−0.015 (11)	−0.023 (7)
F3X	0.126 (9)	0.094 (6)	0.048 (3)	−0.055 (5)	0.000 (5)	0.015 (3)
C9	0.0396 (14)	0.0378 (14)	0.0508 (16)	0.0084 (11)	0.0056 (12)	0.0141 (12)
C10	0.0394 (14)	0.0407 (15)	0.0544 (17)	0.0117 (12)	0.0072 (12)	0.0102 (12)
C11	0.0588 (18)	0.0424 (16)	0.0493 (17)	0.0145 (13)	0.0102 (13)	0.0100 (13)
C12	0.0495 (16)	0.0392 (15)	0.0571 (18)	0.0056 (12)	−0.0043 (13)	0.0060 (13)
C13	0.0400 (14)	0.0403 (15)	0.0596 (18)	0.0019 (12)	0.0045 (12)	0.0162 (13)
C14	0.0546 (17)	0.0437 (16)	0.0561 (18)	0.0146 (13)	−0.0041 (14)	0.0010 (13)
C15	0.0458 (16)	0.0470 (17)	0.0532 (17)	0.0145 (13)	0.0115 (13)	0.0094 (13)
C16	0.0424 (15)	0.0432 (16)	0.0544 (17)	0.0123 (12)	0.0019 (12)	0.0140 (12)
C17	0.0567 (18)	0.0427 (16)	0.0560 (17)	0.0136 (13)	0.0108 (14)	0.0193 (13)
C18	0.0386 (15)	0.0353 (15)	0.076 (2)	0.0027 (12)	0.0068 (14)	0.0112 (13)

### Geometric parameters (Å, °)

**Table:**

Fe1—C9	2.027 (3)	C8—F3X	1.260 (10)
Fe1—C10	2.038 (3)	C8—F1X	1.284 (14)
Fe1—C13	2.037 (3)	C8—F1	1.288 (11)
Fe1—C15	2.038 (3)	C8—F2	1.348 (8)
Fe1—C16	2.039 (3)	C8—F3	1.372 (13)
Fe1—C14	2.041 (3)	F2X—F3X	1.44 (4)
Fe1—C17	2.043 (3)	C9—C10	1.434 (4)
Fe1—C11	2.049 (3)	C9—C13	1.438 (4)
Fe1—C12	2.050 (3)	C10—C11	1.412 (4)
Fe1—C18	2.054 (3)	C10—H10	0.9500
C1—N1	1.284 (3)	C11—C12	1.424 (4)
C1—C9	1.446 (4)	C11—H11	0.9500
C1—H1	0.9500	C12—C13	1.408 (4)
N1—C2	1.413 (4)	C12—H12	0.9500
C2—C7	1.388 (4)	C13—H13	0.9500
C2—C3	1.400 (4)	C14—C18	1.416 (4)
C3—C4	1.386 (4)	C14—C15	1.420 (4)
C3—H3	0.9500	C14—H14	0.9500
C4—C5	1.385 (4)	C15—C16	1.418 (4)
C4—H4	0.9500	C15—H15	0.9500
C5—C6	1.387 (4)	C16—C17	1.419 (4)
C5—C8	1.496 (4)	C16—H16	0.9500
C6—C7	1.381 (4)	C17—C18	1.404 (4)
C6—H6	0.9500	C17—H17	0.9500
C7—H7	0.9500	C18—H18	0.9500
C8—F2X	1.213 (12)		
			
C9—Fe1—C10	41.32 (11)	F1X—C8—F2	103.8 (11)
C9—Fe1—C13	41.45 (11)	F1—C8—F2	85.3 (10)
C10—Fe1—C13	69.23 (11)	F2X—C8—F3	107 (2)
C9—Fe1—C15	119.87 (12)	F3X—C8—F3	38.2 (9)
C10—Fe1—C15	155.72 (12)	F1X—C8—F3	77.4 (10)
C13—Fe1—C15	106.89 (12)	F1—C8—F3	98.7 (10)
C9—Fe1—C16	155.40 (12)	F2—C8—F3	131.5 (11)
C10—Fe1—C16	162.11 (11)	F2X—C8—C5	116.2 (7)
C13—Fe1—C16	120.16 (11)	F3X—C8—C5	114.9 (5)
C15—Fe1—C16	40.69 (12)	F1X—C8—C5	114.7 (7)
C9—Fe1—C14	106.89 (12)	F1—C8—C5	112.6 (7)
C10—Fe1—C14	120.63 (12)	F2—C8—C5	112.7 (5)
C13—Fe1—C14	124.95 (12)	F3—C8—C5	110.0 (6)
C15—Fe1—C14	40.75 (11)	C8—F2X—F3X	55.8 (11)
C16—Fe1—C14	68.29 (12)	C8—F3X—F2X	52.8 (9)
C9—Fe1—C17	161.88 (12)	C10—C9—C13	107.4 (2)
C10—Fe1—C17	125.05 (11)	C10—C9—C1	128.3 (2)
C13—Fe1—C17	155.74 (12)	C13—C9—C1	124.1 (2)
C15—Fe1—C17	68.32 (12)	C10—C9—Fe1	69.78 (15)
C16—Fe1—C17	40.68 (11)	C13—C9—Fe1	69.64 (15)
C14—Fe1—C17	67.88 (12)	C1—C9—Fe1	121.78 (19)
C9—Fe1—C11	68.69 (12)	C11—C10—C9	107.8 (2)
C10—Fe1—C11	40.42 (12)	C11—C10—Fe1	70.21 (17)
C13—Fe1—C11	68.35 (12)	C9—C10—Fe1	68.91 (14)
C15—Fe1—C11	162.24 (12)	C11—C10—H10	126.1
C16—Fe1—C11	125.61 (12)	C9—C10—H10	126.1
C14—Fe1—C11	155.91 (13)	Fe1—C10—H10	126.3
C17—Fe1—C11	108.56 (12)	C10—C11—C12	108.5 (3)
C9—Fe1—C12	68.79 (11)	C10—C11—Fe1	69.37 (16)
C10—Fe1—C12	68.55 (11)	C12—C11—Fe1	69.71 (16)
C13—Fe1—C12	40.31 (12)	C10—C11—H11	125.7
C15—Fe1—C12	124.85 (12)	C12—C11—H11	125.7
C16—Fe1—C12	107.87 (12)	Fe1—C11—H11	126.8
C14—Fe1—C12	161.84 (13)	C13—C12—C11	108.3 (3)
C17—Fe1—C12	121.59 (12)	C13—C12—Fe1	69.33 (16)
C11—Fe1—C12	40.65 (12)	C11—C12—Fe1	69.64 (16)
C9—Fe1—C18	124.84 (12)	C13—C12—H12	125.9
C10—Fe1—C18	107.73 (11)	C11—C12—H12	125.9
C13—Fe1—C18	162.30 (13)	Fe1—C12—H12	126.7
C15—Fe1—C18	68.31 (12)	C12—C13—C9	108.0 (2)
C16—Fe1—C18	68.09 (11)	C12—C13—Fe1	70.36 (16)
C14—Fe1—C18	40.46 (12)	C9—C13—Fe1	68.90 (15)
C17—Fe1—C18	40.09 (12)	C12—C13—H13	126.0
C11—Fe1—C18	121.37 (12)	C9—C13—H13	126.0
C12—Fe1—C18	156.31 (13)	Fe1—C13—H13	126.3
N1—C1—C9	123.8 (2)	C18—C14—C15	108.2 (3)
N1—C1—H1	118.1	C18—C14—Fe1	70.27 (16)
C9—C1—H1	118.1	C15—C14—Fe1	69.53 (16)
C1—N1—C2	118.0 (2)	C18—C14—H14	125.9
C7—C2—C3	118.9 (3)	C15—C14—H14	125.9
C7—C2—N1	117.4 (3)	Fe1—C14—H14	125.9
C3—C2—N1	123.6 (3)	C16—C15—C14	107.6 (3)
C4—C3—C2	120.0 (3)	C16—C15—Fe1	69.69 (16)
C4—C3—H3	120.0	C14—C15—Fe1	69.72 (16)
C2—C3—H3	120.0	C16—C15—H15	126.2
C3—C4—C5	120.4 (3)	C14—C15—H15	126.2
C3—C4—H4	119.8	Fe1—C15—H15	126.0
C5—C4—H4	119.8	C15—C16—C17	107.8 (3)
C4—C5—C6	120.0 (3)	C15—C16—Fe1	69.62 (17)
C4—C5—C8	120.8 (3)	C17—C16—Fe1	69.82 (16)
C6—C5—C8	119.2 (3)	C15—C16—H16	126.1
C7—C6—C5	119.7 (3)	C17—C16—H16	126.1
C7—C6—H6	120.2	Fe1—C16—H16	126.0
C5—C6—H6	120.2	C18—C17—C16	108.5 (3)
C6—C7—C2	121.1 (3)	C18—C17—Fe1	70.35 (17)
C6—C7—H7	119.4	C16—C17—Fe1	69.50 (16)
C2—C7—H7	119.4	C18—C17—H17	125.8
F2X—C8—F3X	71.3 (19)	C16—C17—H17	125.8
F2X—C8—F1X	122.9 (13)	Fe1—C17—H17	126.0
F3X—C8—F1X	108.1 (9)	C17—C18—C14	107.9 (2)
F2X—C8—F1	110.8 (16)	C17—C18—Fe1	69.56 (16)
F3X—C8—F1	124.4 (8)	C14—C18—Fe1	69.28 (16)
F1X—C8—F1	22.1 (12)	C17—C18—H18	126.0
F2X—C8—F2	31.8 (15)	C14—C18—H18	126.0
F3X—C8—F2	101.3 (9)	Fe1—C18—H18	126.7
			
C9—C1—N1—C2	−178.9 (2)	C18—Fe1—C12—C11	−48.3 (3)
C1—N1—C2—C7	−141.5 (3)	C11—C12—C13—C9	−0.1 (3)
C1—N1—C2—C3	39.9 (4)	Fe1—C12—C13—C9	58.81 (19)
C7—C2—C3—C4	1.9 (4)	C11—C12—C13—Fe1	−58.9 (2)
N1—C2—C3—C4	−179.5 (2)	C10—C9—C13—C12	0.1 (3)
C2—C3—C4—C5	−0.6 (4)	C1—C9—C13—C12	−175.0 (3)
C3—C4—C5—C6	−0.7 (4)	Fe1—C9—C13—C12	−59.7 (2)
C3—C4—C5—C8	−179.2 (3)	C10—C9—C13—Fe1	59.83 (18)
C4—C5—C6—C7	0.8 (4)	C1—C9—C13—Fe1	−115.3 (3)
C8—C5—C6—C7	179.3 (3)	C9—Fe1—C13—C12	119.3 (2)
C5—C6—C7—C2	0.5 (4)	C10—Fe1—C13—C12	80.94 (18)
C3—C2—C7—C6	−1.8 (4)	C15—Fe1—C13—C12	−124.41 (18)
N1—C2—C7—C6	179.4 (2)	C16—Fe1—C13—C12	−82.1 (2)
C4—C5—C8—F2X	14.3 (19)	C14—Fe1—C13—C12	−165.50 (16)
C6—C5—C8—F2X	−164.2 (19)	C17—Fe1—C13—C12	−49.8 (3)
C4—C5—C8—F3X	94.9 (9)	C11—Fe1—C13—C12	37.44 (17)
C6—C5—C8—F3X	−83.7 (9)	C18—Fe1—C13—C12	164.2 (3)
C4—C5—C8—F1X	−139.0 (12)	C10—Fe1—C13—C9	−38.38 (16)
C6—C5—C8—F1X	42.5 (12)	C15—Fe1—C13—C9	116.27 (17)
C4—C5—C8—F1	−114.9 (8)	C16—Fe1—C13—C9	158.53 (16)
C6—C5—C8—F1	66.5 (9)	C14—Fe1—C13—C9	75.2 (2)
C4—C5—C8—F2	−20.5 (7)	C17—Fe1—C13—C9	−169.2 (2)
C6—C5—C8—F2	161.0 (6)	C11—Fe1—C13—C9	−81.87 (18)
C4—C5—C8—F3	136.1 (14)	C12—Fe1—C13—C9	−119.3 (2)
C6—C5—C8—F3	−42.5 (14)	C18—Fe1—C13—C9	44.9 (4)
F1X—C8—F2X—F3X	−99.9 (19)	C9—Fe1—C14—C18	−124.34 (17)
F1—C8—F2X—F3X	−120.7 (11)	C10—Fe1—C14—C18	−81.4 (2)
F2—C8—F2X—F3X	−160 (2)	C13—Fe1—C14—C18	−166.32 (17)
F3—C8—F2X—F3X	−14.1 (12)	C15—Fe1—C14—C18	119.2 (3)
C5—C8—F2X—F3X	109.2 (9)	C16—Fe1—C14—C18	81.25 (18)
F1X—C8—F3X—F2X	119.5 (12)	C17—Fe1—C14—C18	37.24 (17)
F1—C8—F3X—F2X	102.8 (12)	C11—Fe1—C14—C18	−48.9 (4)
F2—C8—F3X—F2X	10.8 (11)	C12—Fe1—C14—C18	162.4 (3)
F3—C8—F3X—F2X	157.8 (16)	C9—Fe1—C14—C15	116.43 (18)
C5—C8—F3X—F2X	−110.9 (10)	C10—Fe1—C14—C15	159.34 (17)
N1—C1—C9—C10	8.2 (4)	C13—Fe1—C14—C15	74.4 (2)
N1—C1—C9—C13	−177.7 (3)	C16—Fe1—C14—C15	−37.99 (17)
N1—C1—C9—Fe1	96.6 (3)	C17—Fe1—C14—C15	−81.99 (19)
C13—Fe1—C9—C10	−118.5 (2)	C11—Fe1—C14—C15	−168.1 (2)
C15—Fe1—C9—C10	159.84 (16)	C12—Fe1—C14—C15	43.1 (4)
C16—Fe1—C9—C10	−167.9 (2)	C18—Fe1—C14—C15	−119.2 (3)
C14—Fe1—C9—C10	117.46 (17)	C18—C14—C15—C16	−0.2 (3)
C17—Fe1—C9—C10	47.2 (4)	Fe1—C14—C15—C16	59.6 (2)
C11—Fe1—C9—C10	−37.45 (16)	C18—C14—C15—Fe1	−59.9 (2)
C12—Fe1—C9—C10	−81.22 (17)	C9—Fe1—C15—C16	160.10 (16)
C18—Fe1—C9—C10	76.70 (19)	C10—Fe1—C15—C16	−166.3 (2)
C10—Fe1—C9—C13	118.5 (2)	C13—Fe1—C15—C16	116.89 (17)
C15—Fe1—C9—C13	−81.71 (19)	C14—Fe1—C15—C16	−118.7 (2)
C16—Fe1—C9—C13	−49.5 (3)	C17—Fe1—C15—C16	−37.88 (17)
C14—Fe1—C9—C13	−124.09 (17)	C11—Fe1—C15—C16	45.3 (4)
C17—Fe1—C9—C13	165.6 (3)	C12—Fe1—C15—C16	76.3 (2)
C11—Fe1—C9—C13	81.00 (18)	C18—Fe1—C15—C16	−81.18 (18)
C12—Fe1—C9—C13	37.23 (17)	C9—Fe1—C15—C14	−81.2 (2)
C18—Fe1—C9—C13	−164.85 (17)	C10—Fe1—C15—C14	−47.6 (3)
C10—Fe1—C9—C1	−123.3 (3)	C13—Fe1—C15—C14	−124.39 (18)
C13—Fe1—C9—C1	118.2 (3)	C16—Fe1—C15—C14	118.7 (2)
C15—Fe1—C9—C1	36.5 (3)	C17—Fe1—C15—C14	80.83 (19)
C16—Fe1—C9—C1	68.8 (3)	C11—Fe1—C15—C14	164.0 (3)
C14—Fe1—C9—C1	−5.9 (2)	C12—Fe1—C15—C14	−164.95 (18)
C17—Fe1—C9—C1	−76.1 (4)	C18—Fe1—C15—C14	37.54 (18)
C11—Fe1—C9—C1	−160.8 (2)	C14—C15—C16—C17	0.0 (3)
C12—Fe1—C9—C1	155.5 (2)	Fe1—C15—C16—C17	59.63 (19)
C18—Fe1—C9—C1	−46.6 (3)	C14—C15—C16—Fe1	−59.7 (2)
C13—C9—C10—C11	−0.1 (3)	C9—Fe1—C16—C15	−45.2 (3)
C1—C9—C10—C11	174.8 (3)	C10—Fe1—C16—C15	161.5 (3)
Fe1—C9—C10—C11	59.68 (19)	C13—Fe1—C16—C15	−80.76 (19)
C13—C9—C10—Fe1	−59.74 (18)	C14—Fe1—C16—C15	38.04 (17)
C1—C9—C10—Fe1	115.1 (3)	C17—Fe1—C16—C15	118.9 (2)
C9—Fe1—C10—C11	−119.1 (2)	C11—Fe1—C16—C15	−164.53 (16)
C13—Fe1—C10—C11	−80.61 (18)	C12—Fe1—C16—C15	−123.09 (17)
C15—Fe1—C10—C11	−165.7 (2)	C18—Fe1—C16—C15	81.77 (18)
C16—Fe1—C10—C11	44.4 (4)	C9—Fe1—C16—C17	−164.1 (2)
C14—Fe1—C10—C11	160.21 (17)	C10—Fe1—C16—C17	42.6 (4)
C17—Fe1—C10—C11	77.1 (2)	C13—Fe1—C16—C17	160.32 (16)
C12—Fe1—C10—C11	−37.27 (17)	C15—Fe1—C16—C17	−118.9 (2)
C18—Fe1—C10—C11	117.87 (18)	C14—Fe1—C16—C17	−80.88 (19)
C13—Fe1—C10—C9	38.50 (16)	C11—Fe1—C16—C17	76.6 (2)
C15—Fe1—C10—C9	−46.6 (3)	C12—Fe1—C16—C17	118.00 (18)
C16—Fe1—C10—C9	163.6 (3)	C18—Fe1—C16—C17	−37.15 (18)
C14—Fe1—C10—C9	−80.67 (19)	C15—C16—C17—C18	0.3 (3)
C17—Fe1—C10—C9	−163.83 (16)	Fe1—C16—C17—C18	59.8 (2)
C11—Fe1—C10—C9	119.1 (2)	C15—C16—C17—Fe1	−59.5 (2)
C12—Fe1—C10—C9	81.85 (17)	C9—Fe1—C17—C18	38.9 (4)
C18—Fe1—C10—C9	−123.01 (17)	C10—Fe1—C17—C18	75.2 (2)
C9—C10—C11—C12	0.0 (3)	C13—Fe1—C17—C18	−164.7 (2)
Fe1—C10—C11—C12	58.9 (2)	C15—Fe1—C17—C18	−81.65 (18)
C9—C10—C11—Fe1	−58.87 (18)	C16—Fe1—C17—C18	−119.5 (2)
C9—Fe1—C11—C10	38.25 (16)	C14—Fe1—C17—C18	−37.57 (17)
C13—Fe1—C11—C10	82.96 (18)	C11—Fe1—C17—C18	116.99 (18)
C15—Fe1—C11—C10	160.6 (3)	C12—Fe1—C17—C18	159.84 (17)
C16—Fe1—C11—C10	−164.66 (16)	C9—Fe1—C17—C16	158.5 (3)
C14—Fe1—C11—C10	−45.5 (4)	C10—Fe1—C17—C16	−165.28 (16)
C17—Fe1—C11—C10	−122.69 (17)	C13—Fe1—C17—C16	−45.1 (3)
C12—Fe1—C11—C10	120.1 (2)	C15—Fe1—C17—C16	37.89 (17)
C18—Fe1—C11—C10	−80.5 (2)	C14—Fe1—C17—C16	81.96 (18)
C9—Fe1—C11—C12	−81.84 (18)	C11—Fe1—C17—C16	−123.48 (18)
C10—Fe1—C11—C12	−120.1 (2)	C12—Fe1—C17—C16	−80.6 (2)
C13—Fe1—C11—C12	−37.14 (17)	C18—Fe1—C17—C16	119.5 (3)
C15—Fe1—C11—C12	40.5 (4)	C16—C17—C18—C14	−0.4 (3)
C16—Fe1—C11—C12	75.2 (2)	Fe1—C17—C18—C14	58.8 (2)
C14—Fe1—C11—C12	−165.6 (3)	C16—C17—C18—Fe1	−59.2 (2)
C17—Fe1—C11—C12	117.21 (18)	C15—C14—C18—C17	0.4 (3)
C18—Fe1—C11—C12	159.45 (17)	Fe1—C14—C18—C17	−59.0 (2)
C10—C11—C12—C13	0.1 (3)	C15—C14—C18—Fe1	59.4 (2)
Fe1—C11—C12—C13	58.7 (2)	C9—Fe1—C18—C17	−166.23 (16)
C10—C11—C12—Fe1	−58.7 (2)	C10—Fe1—C18—C17	−123.81 (17)
C9—Fe1—C12—C13	−38.26 (17)	C13—Fe1—C18—C17	159.1 (3)
C10—Fe1—C12—C13	−82.77 (18)	C15—Fe1—C18—C17	81.67 (18)
C15—Fe1—C12—C13	74.1 (2)	C16—Fe1—C18—C17	37.69 (17)
C16—Fe1—C12—C13	115.86 (18)	C14—Fe1—C18—C17	119.5 (2)
C14—Fe1—C12—C13	41.2 (4)	C11—Fe1—C18—C17	−81.6 (2)
C17—Fe1—C12—C13	158.37 (17)	C12—Fe1—C18—C17	−46.9 (3)
C11—Fe1—C12—C13	−119.8 (3)	C9—Fe1—C18—C14	74.3 (2)
C18—Fe1—C12—C13	−168.1 (2)	C10—Fe1—C18—C14	116.72 (18)
C9—Fe1—C12—C11	81.58 (18)	C13—Fe1—C18—C14	39.6 (4)
C10—Fe1—C12—C11	37.07 (17)	C15—Fe1—C18—C14	−37.81 (17)
C13—Fe1—C12—C11	119.8 (3)	C16—Fe1—C18—C14	−81.79 (18)
C15—Fe1—C12—C11	−166.04 (16)	C17—Fe1—C18—C14	−119.5 (2)
C16—Fe1—C12—C11	−124.31 (18)	C11—Fe1—C18—C14	158.89 (17)
C14—Fe1—C12—C11	161.0 (3)	C12—Fe1—C18—C14	−166.4 (2)
C17—Fe1—C12—C11	−81.8 (2)		
